# A bibliometric study of global trends in T1DM and intestinal flora research

**DOI:** 10.3389/fmicb.2024.1403514

**Published:** 2024-07-04

**Authors:** Xinxin Cui, Zhen Wu, Yangbo Zhou, Longji Deng, Yu Chen, Hanqiao Huang, Xiangbin Sun, Yu Li, Haixia Wang, Li Zhang, Jia He

**Affiliations:** ^1^Department of Public Health, Shihezi University School of Medicine, Shihezi, Xinjiang, China; ^2^Key Laboratory for Prevention and Control of Emerging Infectious Diseases and Public Health Security, The Xinjiang Production and Construction Corps, Shihezi, Xinjiang, China; ^3^Department of Public Health and Key Laboratory of Xinjiang Endemic and Ethnic Diseases of the Ministry of Education, School of Medicine, Shihezi University, Shihezi, Xinjiang, China

**Keywords:** T1DM, intestinal flora, bibliometrics, CiteSpace, VOSviewers

## Abstract

**Background:**

Type 1 diabetes mellitus (T1DM) is a chronic metabolic disease that seriously jeopardizes human physical and mental health and reduces quality of life. Intestinal flora is one of the critical areas of exploration in T1DM research.

**Objective:**

This study aims to explore the research hotspot and development trend of T1DM and intestinal flora to provide research direction and ideas for researchers.

**Methods:**

We used the Web of Science (WOS) Core Collection and searched up to 18 November 2023, for articles on studies of the correlation between T1DM and intestinal flora. CiteSpace, VOSviewers and R package “bibliometrix” were used to conduct this bibliometric analysis.

**Results:**

Eventually, 534 documents met the requirements to be included, and as of 18 November 2023, there was an upward trend in the number of publications in the field, with a significant increase in the number of articles published after 2020. In summary, F Susan Wong (UK) was the author with the most publications (21), the USA was the country with the most publications (198), and the State University System of Florida (the United States) was the institution with the most publications (32). The keywords that appeared more frequently were T cells, fecal transplants, and short-chain fatty acids. The results of keywords with the most robust citation bursts suggest that *Faecalibacterium prausnitzii* and butyrate may become a focus of future research.

**Conclusion:**

In the future, intestinal flora will remain a research focus in T1DM. Future research can start from *Faecalibacterium prausnitzii* and combine T cells, fecal bacteria transplantation, and short-chain fatty acids to explore the mechanism by which intestinal flora affects blood glucose in patients with T1DM, which may provide new ideas for the prevention and treatment of T1DM.

## 1 Introduction

Type 1 diabetes mellitus (T1DM) is a metabolic disorders caused by autoimmunity leading to a lack of insulin secretion from pancreatic beta cells (Zhang et al., 2022a). According to data published by the International Diabetes Federation (IDF) in 2021, approximately 1.2 million people worldwide with T1DM were adolescents and children (Saeedi et al., 2019). The low age and poor cognition of most T1DM patients at the onset of the disease make glycemic control very difficult and complication rates high, seriously jeopardizing the lives and health of patients (Bruno et al., 2013; Katsarou et al., 2017; Li et al., 2022; Riveline et al., 2022). The pathogenesis of T1DM is genetic, and non-genetic factors play an increasingly important role in it (Christovich and Luo, 2022; Zhang et al., 2022c). In recent years, there has been a growing interest in non-genetic factors in intestinal flora changes such as the pathogenesis of T1DM (Del Chierico et al., 2022). The intestinal flora of patients with T1DM differs from that of healthy individuals. For instance, the Firmicutes to Bacteroidetes ratio significantly decreased, with the quantity of Bacteroidetes significantly increasing for healthy children (Murri et al., 2013). Intestinal flora affected host metabolism and immune network activity, ultimately influencing diabetes development (Musso et al., 2011). In addition, intestinal flora also plays an important role in the complications of T1DM. T1DM patients with depression have a higher abundance of Megasphaera than those without depression (Liu et al., 2024). Moreover, the metabolites of intestinal flora also play an important role in T1DM. The tryptophan metabolites mediated by intestinal flora are an important marker for distinguishing between rapid and chronic progressors of T1DM (Lamichhane et al., 2023). The phenolic acids produced by beneficial bacteria have high antioxidant, anti-diabetic, and anti-inflammatory properties, which can accelerate T cell differentiation, increase the number of Treg, and inhibit the release of inflammatory cytokines in macrophages (Hu et al., 2023). Most patients with type 1 diabetes do not have a family history. Low genetic risk does not rule out the development of clinical type 1 diabetes. Ruminococcus is a powerful determinant for distinguishing control infants from future type 1 diabetes infants in a random forest analysis (Bélteky et al., 2023). Current research has found that the results of T1DM and intestinal flora in terms of α and β diversity are inconsistent (Traversi et al., 2022; Flasbeck et al., 2023). In addition, current research focuses on bacteria, with few studies exploring fungi and viruses. Coxsackievirus B infection is considered to be a modulator of beta cell autoimmunity, which may be developed into a vaccine to reduce the incidence of T1DM (Traversi et al., 2022). Therefore, exploring the intestinal flora is essential for studying the mechanism of T1DM. In the past, many scholars focused on exploring the mechanisms and therapeutic means for the occurrence of T1DM with the intestinal flora, including intestinal permeability. Many cutting-edge technological approaches, such as macro-genomics and metabolomics, have also been applied to this field (Stewart et al., 2018). However, the specific mechanisms between T1DM and intestinal flora and new research hotspots remain to be explored.

Through statistical techniques, bibliometric analysis allows for analyzing research trends (Zhu et al., 2021). The methodology has been created and applied to various industries, including studies in analytical chemistry, environmental studies, and epidemiology (Fu et al., 2022; Yang et al., 2022; Zhong et al., 2022). Currently, we found only one bibliometric study on T1DM and intestinal flora. Zhang et al.’s (2022b) study only selected the literature on diabetes and intestinal flora correlation studies conducted between 2011 and 2021, with a collection timeframe of only ten years and a discussion focusing on Type 2 Diabetes Mellitus. Therefore, this study conducted a bibliometric analysis based on the records of literature published up to 18 November 2023, including the year, keywords, authors, country, institution, and co-citations of the relevant research publications, to identify the research hotspots and trends in the field, and to provide a reference for future research and applications in the field of T1DM and intestinal flora.

## 2 Materials and methods

### 2.1 Data source

The original data for this study were obtained from the Web of Science (WOS) Core Collection, searched up to 18 November 2023, for literature published during the period.

### 2.2 Data search strategy

To obtain as comprehensive data as possible, this study was searched with the help of the Medical Subject Headings (MeSHs), using T1DM AND intestinal flora and its upstream and downstream terms as the subject terms according to the search rules of Web of Science. In the Supplementary material, specific search tactics were given.

### 2.3 Data extraction and collection

The type of literature included in this study only had research articles and reviews, and the search language was limited to English. The screening of this study was carried out independently by two researchers simultaneously. A total of 2,134 records were searched, and the titles, abstracts, and keywords were read to determine whether the content had a clear association with “T1DM” or “intestinal flora” and then manually filtered. If these were insufficient to determine whether there was a clear correlation between “T1DM and intestinal flora,” the full text was reviewed. The two researchers retrieved 547 and 517 articles after de-duplication and exclusion of literature that did not meet the inclusion criteria. The results of the inclusion analyses were exchanged, and the third researcher decided to include those articles that were difficult to have due to disagreement. In the end, 534 articles met the inclusion criteria. The flow chart of the study is shown in Supplementary Figure 1.

### 2.4 Bibliometric analysis

The 534 documents that met the criteria were selected for the study, exported and downloaded as Plain text files, Tab delimited files and BibTeX, and the output journals and authors were identified using VOSviewers (1.6.18) to construct a relevant network of major co-cited journals and authors. Keywords, countries, institutions, and journals were extracted and organized according to the frequency of occurrence using CiteSpace (6.1.R3), and co-occurrence mapping was generated. The R package “bibliometrix” (version 4.3.1) was applied to visualize the geographical location and cooperation of countries. Annual articles and co-citations were managed using Microsoft Excel (2019) to collect data and analyze publication trends. Ethical approval was not applicable for the present study.

## 3 Results

### 3.1 Publication outputs and trends

**FIGURE 1:**
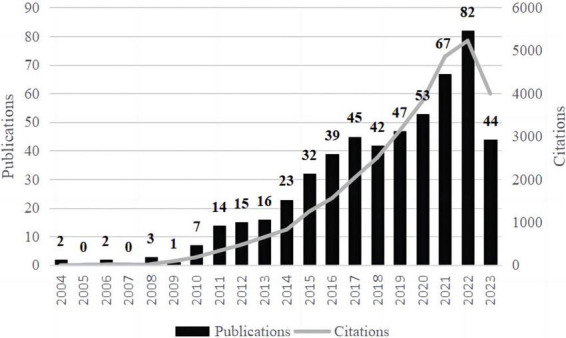
Trends in posting volume and citation frequency annually. The broken line represented the paper’s citation frequency, and the bar graph represented the number of publications of the article.

The current study included 534 on T1DM and intestinal flora up to 18 November 2023. As shown in Figure 1, the number of publications was low from 2004 to 2010, all of which were ten or less. From 2010 onward, the number of articles in this field gradually increased, and studies related to intestinal flora and T1DM received widespread attention. The number of citations in this field in the WoSCC peaked in 2022.

### 3.2 Analysis of journals output

Studies on the correlation between T1DM and intestinal flora were published in 242 journals. In the accompanying Supplementary Figure 2, The dual-map overlay of journals was superimposed to show the distribution of topics. Supplementary Figure 3 depicted the association between journals and time. The journals that have been active in publishing articles in this field in the last five years are Frontiers in Immunology (IF: 7.3), International Journal of Molecular Sciences (IF: 5.6), and so on, which were in the top ten journals in terms of the number of articles issued. The top ten journals regarding the number of reports issued were shown in Table 1, all of which were in Journal Citation Report (JCR)^1^ Q1 and Q2. The journal with the highest impact factor is Diabetologia (IF: 8.2), followed by Diabetes (IF: 7.7).

**TABLE 1:** The top ten most productive journals.

Ranking	Journal	Output	% of 510	IF 2022	JCR 2022
1	Frontiers in Immunology	35	6.554	7.3	Q1
2	PLoS One	19	3.558	3.7	Q2
3	Diabetologia	18	3.371	8.2	Q1
4	Diabetes	15	2.809	7.7	Q1
5	International Journal of Molecular Sciences	14	2.622	5.6	Q1
6	Frontiers in Endocrinology	11	2.060	5.2	Q1
7	Frontiers in Microbiology	11	2.060	5.2	Q2
8	Pediatric Diabetes	11	2.060	3.4	Q3
9	Scientific Reports	11	2.060	4.6	Q2
10	Frontiers in Nutrition	9	1.685	5.0	Q2

### 3.3 Analysis of country cooperation

**FIGURE 2:**
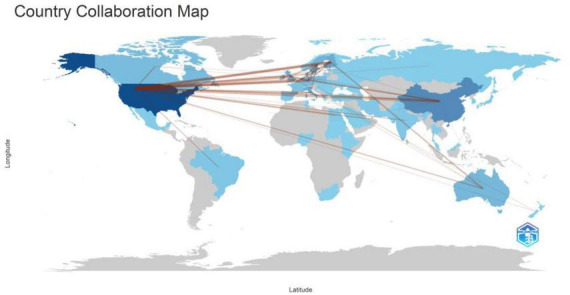
The geographical distribution on research of T1DM and intestinal flora.

**FIGURE 3:**
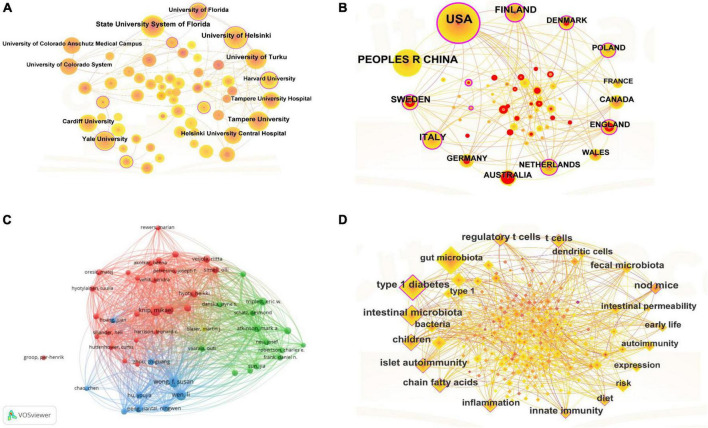
**(A)** Each country was represented as a node, and the node size was proportional to the sum of publications. Different nodes were given different colors according to the color gradient in the lower right corner. **(B)** In this map, a node represented an institution, and the size of each node represented its relative quantity of research output. Each line represented the strength of the cooperation relationship between the two institutions, and strength value was displayed between the lines. **(C)** Each author was represented as a node, and the node size was proportional to the sum of citations. A link between two nodes indicated a co-citation relationship. The distance between nodes indicated the relatedness, and a smaller distance implied a higher relatedness and would be assigned to one cluster with the same colors. **(D)** Each keyword represented a node; the node size was proportional to the number of documents, and the line between the two nodes represented the connection between the keywords.

Studies on T1DM and intestinal flora were conducted in 206 countries or regions. We filtered and visualized these country states and constructed a collaborative network based on the number of publications and relationships in each country (Figure 2). Figure 3A depicted the partnership between individual countries, with the ability of governments to collaborate increasing with increasing centrality. Supplementary Table 1 listed the ten countries with the most published articles. The United States ranks first with 198 pieces, followed by China and Finland, with over 50 publications.

### 3.4 Analysis of institutional cooperation

There were 2,081 institutions involved in research on T1DM and intestinal flora, and the partnerships between institutions were depicted in Figure 3B. The top five institutions in terms of the number of publications were State University System of Florida (32), University of Helsinki (30), University of Turku (26), Tampere University (24) and Yale University (24). Notably, no Chinese institutions were among the top five regarding publications. High-productivity institutions led institutional collaborations, and each institution was well-communicated and closely connected with Yale University or the State University System of Florida and other universities, respectively.

### 3.5 Analysis of author collaboration network

There were 2,796 authors involved in the T1DM and intestinal flora studies. Figure 3C described the authors’ co-citations using VOSviewers (1.6.18). In the visualization map, the authors formed their collaborative teams, and the different colored clusters reflected the collaborative relationship between the authors. In the red group, Knip, Mikael and Lernmark, Ake were the members of the “The Environmental Determinants of Diabetes in Young People” (TEDDY). They have worked primarily with children and have co-authored 75 articles and published in highly rated journals such as the Jama-Journal of the American Medical Association (IF = 120.7) (Aronsson et al., 2019). T1DM genetically susceptible children, higher gluten intake in the first five years was associated with increased risk of celiac disease autoimmunity and celiac disease. In the blue cluster, Wong, F. Susan and Wen, Li have co-authored 106 publications, mostly in mice, and the two have published an article in Nature proposing that the interaction between gut microbes and the innate immune system was a critical epigenetic factor that modifies susceptibility to T1DM (Pearson et al., 2023). The green group needed more collaboration between authors and more publications.

### 3.6 Analysis of co-cited references

Supplementary Table 2 listed the 15 most cited articles. Among them, in terms of research methodology, four pieces used single-omics analyses, such as macro-genomics and metabolomics, and five parts used multiple-omics analyses. In terms of study design, three teams designed case-control studies, and two teams designed cohort studies with sample sizes of 33 and 222, respectively, all in Northern Europe. Two other groups, part of the TEDDY study, created cohort and case-control studies of 903 and 783 children from six clinical centers in four countries (Finland, Germany, Sweden, and the USA).

### 3.7 Analysis of keywords

Figure 3D showed that 20 keywords appeared more than 30 times in this field. In terms of frequency, in addition to the keywords related to the subject terms “intestinal flora” and “T1DM,” the keywords with a frequency of more than 30 and had a high degree of centrality included “T cells,” “short-chain fatty acids,” and “fecal transplants,” and the rest of the keywords were closely related.

Supplementary Table 3 showed the top 35 terms with the most robust citation bursts in the field, butyrate, and *Faecalibacterium prausnitzii* appearing in 2021 and continuing to this day.

## 4 Discussion

In this study, we conducted a systematic bibliometric assessment of the literature on correlation studies between T1DM and intestinal flora from 18 November 2023.

### 4.1 Global publication and journal trends

Changes in the number of academic publications were an essential indicator of the development trend of a field (Peng et al., 2020). With the year-on-year increase in publications and co-citations, we have clarified that studying the correlation between T1DM and intestinal flora has become a research hotspot. The number of literature in the field will increase further with the deepening of the molecular mechanism of T1DM and intestinal flora.

The research on T1DM and intestinal flora was extensive and published in 242 journals. Supplementary Figure 2 showed that literature published in Molecular, Biology, and Genetics journals were cited in the same journals and Medicine, Medical, and Clinical journals with a higher. The high frequency implied that the current research on intestinal flora and T1DM mainly focused on basic medical research. Supplementary Figure 3 showed that articles in this field can be published in journals in multiple areas, such as GUT, MICROBIOME, and NUTRIENTS. Prospective scholars can prioritize research outputs from journals with the highest number of publications and the top three impact factors (Diabetologia, Diabetes, Frontiers in Immunology) to gain access to their cutting-edge information.

### 4.2 Countries, institutions, and authors

Combining Figures 2, 3A, B, There was much optimistic cooperation between the USA, European countries, and China. Among the top five countries in terms of the number of publications, the United States and China have the highest number of publications, and they also have the top five diabetics in the world. The top five institutions in the number of publications are universities in developed countries (Finland and the United States). However, there was no Chinese institution among the top five institutions. This suggested that research on the correlation between T1DM and the intestinal flora required a certain degree of academic atmosphere and financial support. Combined with Figure 3C, the team leaders of each cluster have published in highly rated journals, which researchers can read for easy reference and learning of cutting-edge content. Although the authors within each collection worked closely together and produced a lot of collaborative outputs, the collaboration between team leaders was not strong, and it was expected that researchers could strengthen their cooperation in the future to promote the development of research in the field of T1DM and intestinal flora.

The top-ranked issuing organizations, countries, and authors were more closely linked to each other, suggesting that collaboration would lead to more productive outputs. Meanwhile, analyzing the dynamics of intestinal flora required extensive cohort studies. Individual country studies include the Type 1 Diabetes Prediction and Prevention (DIPP) Study (Nanto-Salonen et al., 2008) and the All Babies in South-East Sweden (ABIS) study (Russell et al., 2019). Multinational studies in several countries include the “The Environmental Determinants of Diabetes in Young People” (TEDDY) study (Stewart et al., 2018; Vatanen et al., 2018), one of the most extensive infant microbiome datasets to date, of which Knip, Mikael, and Lernmark, Ake, who were members of the Red Cluster of the Authors’ Collaborative Network and whose results have also been published in highly rated journals. Collaboration between different countries can collect samples from other regions, integrating different geographic environments and living habits and exploring their effects on the composition of the intestinal flora (Abela and Fava, 2019). However, no extensive birth cohort studies have been conducted in Asian countries such as China or African and South American countries.

### 4.3 References co-citation analysis

The top fifteen cited literature in Supplementary Table 2 were published in journals with high-impact factors. Cohort and case-control studies were the main research methods, and both had advantages and disadvantages. Case-control studies were less costly, but the sample was poorly represented, and care needed to be taken to control the matching of dietary habits, duration of breastfeeding, mode of delivery, and physiological factors such as age, gender, ethnicity, and geographic factors (Murri et al., 2013). Cohort studies were costly and proved to loss of visits. Notably, some researchers combined the design of cohort studies with case-control study methodology and published them in Nature, providing a new idea to explore the relationship between dynamic changes in intestinal flora and T1DM (Stewart et al., 2018).

Researchers were currently using single-omics, such as 16S rRNA sequencing and Metagenome sequencing, for more analytical studies of intestinal flora. 16S rRNA analytical methods usually do not provide a functional genetic composition of the bacterial community. In contrast, Metagenome sequencing studies were particularly well suited to characterize the microbial assemblage at a functional level (Hamady and Knight, 2009). Metabolomics analyses can help researchers understand cellular metabolism and reveal the microbiome’s impact on human health and disease (Joice et al., 2014; Wishart, 2016). The limitation was that metabolomics only provides static information on metabolite abundance. However, researchers have made less use of multi-omics, and little literature has been published on applying lipidomics and proteomics in intestinal flora related to T1DM, which is expected to be developed and used by researchers.

### 4.4 Hot trends and hot changes

Combined with the keyword analysis results in Figure 3D, T cells appeared most frequently (excluding the subject term). Several specific bacteria have been shown to influence T1DM by modulating the phenotypic differentiation of T cells (Krych et al., 2015). Clostridial clusters IV and XIVa, Bacteroides fragilis, and Lactobacillus can generate regulatory T cells (Atarashi et al., 2013). However, the dominant microorganisms associated with T1DM Acetatifactor, Coprococcus_2, or Lachnoclostridium and T cells have not been published and need further investigation in future work. Gazali et al. (2020) used the most extensive and rigorous age-matched cohort study analyzed to demonstrate that peripheral blood MAIT cell alterations were associated with the clinical onset of T1DM. However, their research found reduced expression of β7 integrins on regular CD3 T cells in children with T1DM. Others have not verified this result, suggesting a defect in mucosal T-cell homing in children with T1DM, which should be investigated in more detail in the future (Gazali et al., 2020).

In addition, we found that fecal microbiota transplantation (FMT) has increased in research fervor in recent years. A recently published long-term cohort study showed that FMT had a favorable short- and long-term safety profile without increasing the risk of infection transmission and new-onset disease (Tian et al., 2022). A longitudinal randomized controlled study published by de Groot et al. (2021) showed that when FMT was administered to patients with a recent diagnosis of TIDM, FMT prevented the decline in endogenous insulin secretion in patients after 12 months (de Groot et al., 2021). This was the first report that FMT can affect residual β-cells *in vivo* in patients with new-onset T1DM, and future researchers can continue to add in this direction. However, the ability of the newly established intestinal flora to remain homeostatic for an extended period after performing FMT and its ability to match rather than exacerbate the immune response highly were issues that researchers needed to be concerned about when conducting experiments (Halaweish et al., 2022; Zhang et al., 2022c).

Also appearing at a higher frequency in the keywords were short-chain fatty acids (SCFAs). SCFAs were produced by the fermentation of dietary fiber by intestinal flora, which can suppress the immune response and control the symptoms of T1DM (Wahlstrom et al., 2016). Recent animal studies have shown that altered metabolism of intestinal SCFAs plays a causal role in T1DM (Desai et al., 2016; Marino et al., 2017). Butyrate was one of the main types of SCFAs produced in the colon. Yuan et al. (2022) found in an independent cohort that the microbiota of children with new-onset TIDM was characterized by reduced butyrate production at the species, gene, and metabolite levels. In streptozotocin-induced TIDM mice, butyrate was protective of islet structure and function. Another short-term cohort study had shown that targeting dietary SCFAs may be a mechanism to alter the immune profile, promote immune tolerance, and improve glycemic control for the treatment of T1DM (Bell et al., 2022). However, it has also been demonstrated that oral butyrate treatment in patients with T1DM resulted in a significant decrease in fecal SCFA levels but no change in residual β-cell function, which was inconsistent with findings in the mouse model of T1DM (de Groot et al., 2020). This may be related to the method of administration of butyrate, differences in administration and dosage, differences in pathophysiology between humans and mice, and other aspects. The discrepancy in the results of this study needs to be examined in subsequent studies.

Shilo et al. confirmed that fecal bacterium prausnitzii was one of the most commonly altered intestinal bacterial species in patients with T1DM (Jamshidi et al., 2019; Shilo et al., 2022). A study showed a negative correlation between Faecalibacterium abundance and glycated hemoglobin compared to a healthy population (Rouland et al., 2022). The exact causal mechanism between the two has yet to be reported. The intestinal mucosal changes in T1DM may be related to intestinal inflammation, which may be due to the low abundance of *Faecalibacterium prausnitzii* and other butyrate-producing bacteria (Louis and Flint, 2009). Experimental studies by investigators were needed to determine whether using *Faecalibacterium prausnitzii* as a probiotic had therapeutic or preventive value for intestinal inflammation in patients with T1DM.

## 5 Conclusion

This study used CiteSpace (6.1.R3), VOSviewers (1.6.18) and The R package “bibliometrix” (version 4.3.1) to evaluate 534 literatures as of 18 November 2023 on research on T1DM and intestinal flora. Research on T1DM and intestinal flora was increasing, with 206 countries, 2,081 institutions, and 2,796 authors conducting research in this field. Longitudinal studies with long-term follow-up observations are recommended in this field of research to explore the relationship between T cells, fecal bacterial transplantation, and short-chain fatty acids and to explore the therapeutic value of *Faecalibacterium prausnitzii*, which may provide new ideas for the prevention and treatment of T1DM.

## Data availability statement

The original contributions presented in this study are included in this article/Supplementary material, further inquiries can be directed to the corresponding author.

## Author contributions

XC: Writing – original draft, Writing – review & editing. ZW: Writing – original draft, Writing – review & editing. YZ: Writing – review & editing. LD: Writing – review & editing. YC: Writing – review & editing. HH: Writing – review & editing. XS: Writing – review & editing. YL: Methodology, Project administration, Supervision, Writing – review & editing. HW: Methodology, Project administration, Supervision, Writing – review & editing. LZ: Methodology, Project administration, Supervision, Writing – review & editing. JH: Funding acquisition, Methodology, Project administration, Supervision, Writing – review & editing.

## Footnotes

1
http://clarivate.com/products/web-of-science
