# Supplementary material for: A bibliometric study of global trends in T1DM and intestinal flora research
Source: Front Microbiol. 2024 Jul 4;15:1403514. doi: 10.3389/fmicb.2024.1403514 (PMC11254799; doi:10.3389/fmicb.2024.1403514)
Supplement: Supplementary file 1 [file Table_1.DOCX]

Supplementary Material

(Diabetes Mellitus, Type 1 OR Diabetes Mellitus, Insulin Dependent OR Diabetes Mellitus, Juvenile Onset OR Diabetes Mellitus, Sudden Onset OR Insulin Dependent Diabetes Mellitus 1 OR Diabetes, Type 1 OR Diabetes, Autoimmune OR Wolfram Syndrome)AND(Gastrointestinal Microbiome OR Gastrointestinal Microbiomes OR Microbiome, Gastrointestinal OR Gut Microbiome OR Gut Microbiomes OR Microbiome, Gut OR Gut Microflora OR Microflora, Gut OR Gut Microbiota OR Gut Microbiotas OR Microbiota, Gut OR Gastrointestinal Flora OR Flora, Gastrointestinal OR Gut Flora OR Flora, Gut OR Gastrointestinal Microbiota OR Gastrointestinal Microbiotas OR Microbiota, Gastrointestinal OR Gastrointestinal Microbial Community OR Gastrointestinal Microbial Communities OR Microbial Community, Gastrointestinal OR Gastrointestinal Microflora OR Microflora, Gastrointestinal OR Gastric Microbiome OR Gastric Microbiomes OR Microbiome, Gastric OR Intestinal Microbiome OR Intestinal Microbiomes OR Microbiome, Intestinal OR Intestinal Microbiota OR Intestinal Microbiotas OR Microbiota, Intestinal OR Intestinal Microflora OR Microflora, Intestinal OR Intestinal Flora OR Flora, Intestinal OR Enteric Bacteria OR Bacteria, Enteric)
