# Supplementary material for: A bibliometric study of global trends in T1DM and intestinal flora research
Source: Front Microbiol. 2024 Jul 4;15:1403514. doi: 10.3389/fmicb.2024.1403514 (PMC11254799; doi:10.3389/fmicb.2024.1403514)
Supplement: Supplementary file 2 [file Data_Sheet_1.docx]

Supplementary Material

**supplementary figure 1** **Retrieval process flowchart for the research** 2

**supplementary figure 2 The dual-map overlay of journals in intestinal flora and T1DM**. 3

**supplementary figure 3 Network visualization map of journal co-citation analysis generated by VOSviewer** 4

**supplementary Table 1 The top 10 countries / regions with the most publications** 5

**supplementary Table 2 The top 15 highly cited literature** 6

**supplementary Table 3 The top 35 Keywords outbreak** 7

**Supplementary Figure 1：**Retrieval process flowchart for the research

Publications identified through Web of Science database searching

(WoS Core Collection)

- Retrieval strategy：Diabetes Mellitus* AND Gastrointestinal Microbiome*
- Retrieval time：May 4, 2023
- Retrieval time span: 2004/01/01-2023/05/04.
- Document types: Articles and Reviews
- Language restriction: English

2137 publications were preliminarily identified for further screening

Two independent researchers screened by title, abstract and full text

547 publications

517 publications

The third researcher

534 publications including 336 articles and 174 reviews

Publications, Citations

Journals

Countries

Regions

Authors

Keywords

**
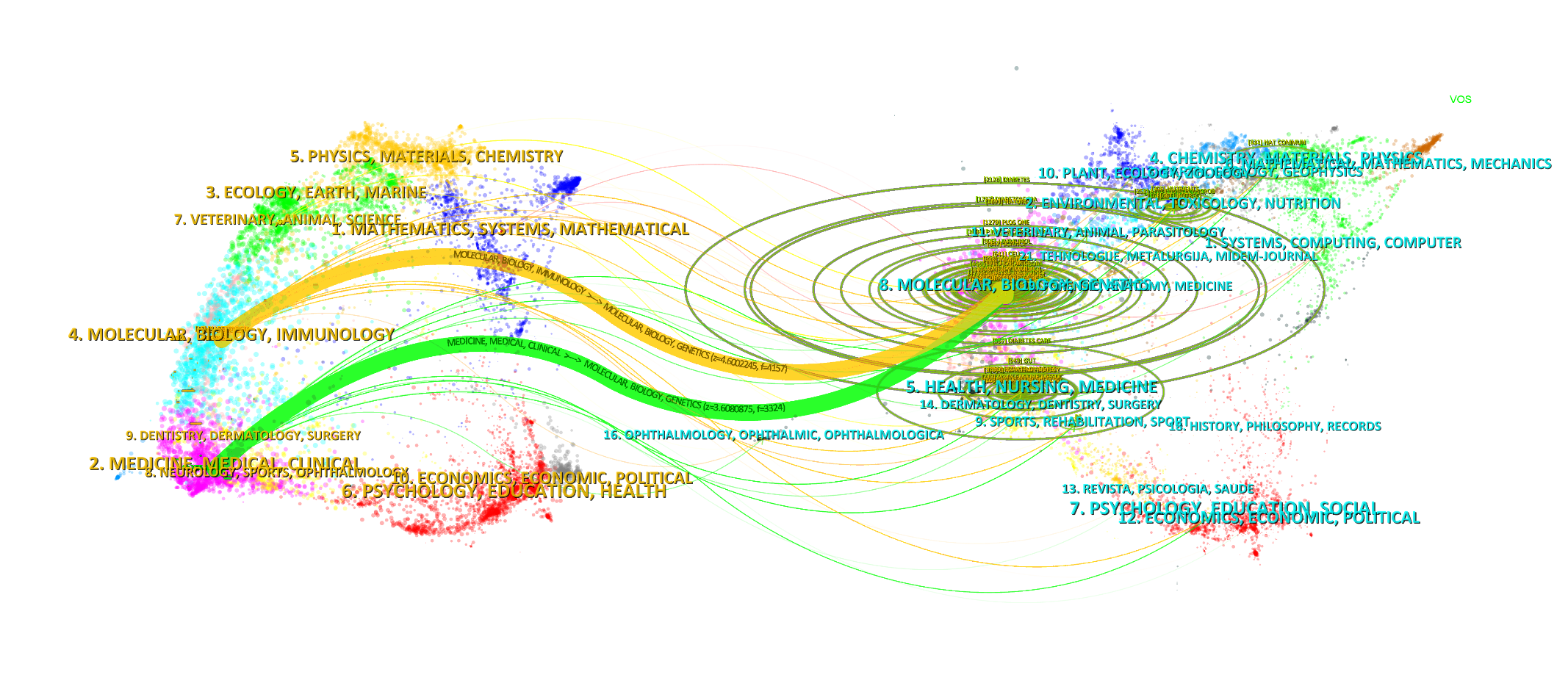
**

###### Supplementary Figure 2 : The dual-map overlay of journals in intestinal flora and T1DM

**Note:** A dual-map overlay of journals showed the distribution of topics. The citing journals were on the left, and the cited journals were on the right. The labels represented the disciplines covered by the journals, and the colored path represented the citation relationship.

######
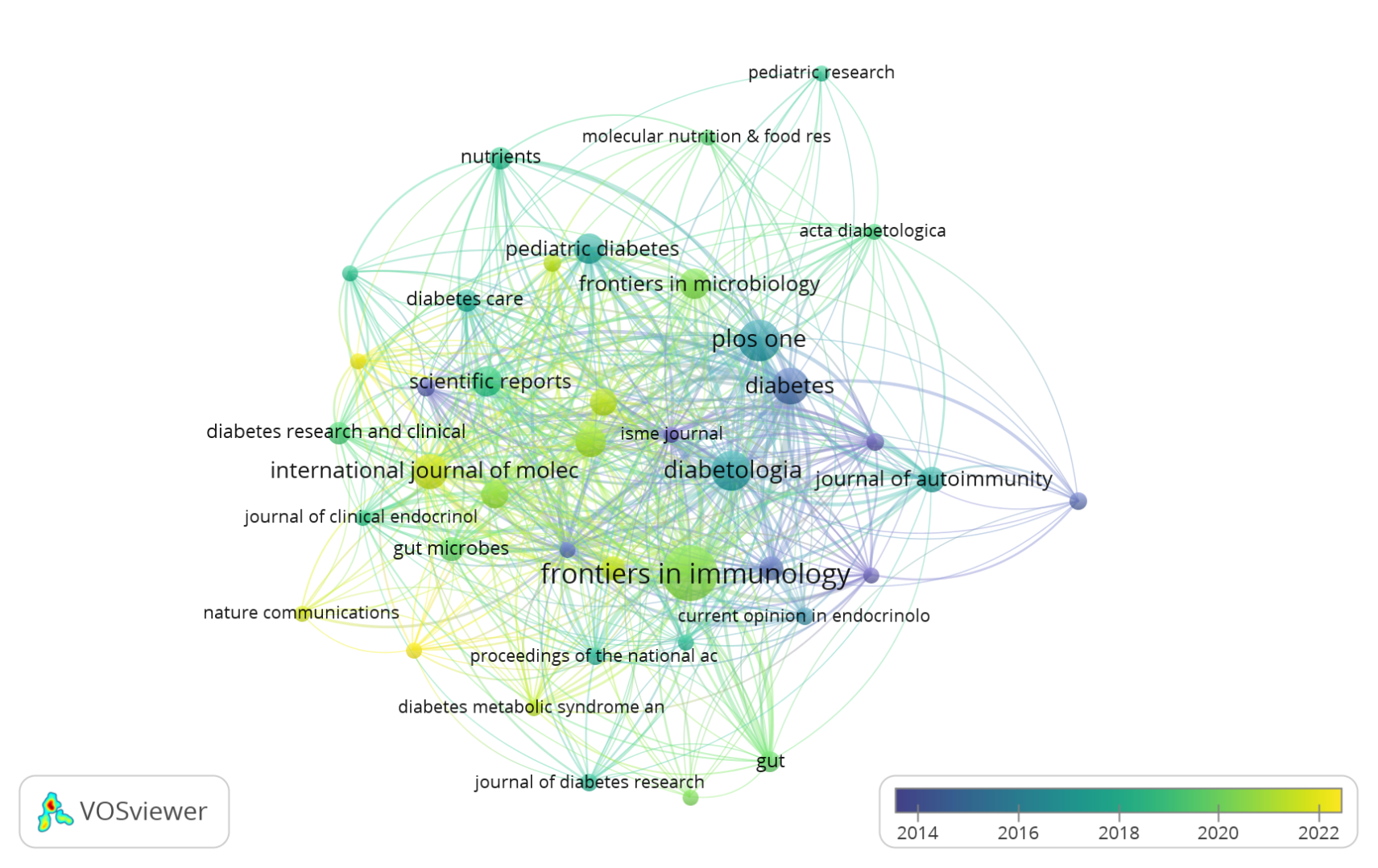


###### Supplementary Figure 3：Network visualization map of journal co-citation analysis generated by VOSviewer.

###### Supplementary Table 1：The top 10 countries / regions with the most publications.

| **Ranking** | **Countries/Area** | **Centrality** | **Year** | **Publications** |
| --- | --- | --- | --- | --- |
| 1 | USA | 0.42 | 2006 | 198 |
| 2 | CHINA | 0.06 | 2014 | 93 |
| 3 | FINLAND | 0.24 | 2008 | 52 |
| 4 | ITALY | 0.11 | 2011 | 46 |
| 5 | SWEDEN | 0.15 | 2004 | 40 |
| 6 | AUSTRALIA | 0.02 | 2013 | 30 |
| 7 | NETHERLANDS | 0.20 | 2006 | 29 |
| 8 | ENGLAND | 0.17 | 2004 | 27 |
| 9 | CANADA | 0.03 | 2010 | 27 |
| 10 | GERMANY | 0.04 | 2013 | 25 |

###### Supplementary Table 2: The top 15 highly cited literature.

| **Title** | **Journals** | **First author** | **Year** | **Citations** | **Citations**/**Year** |
| --- | --- | --- | --- | --- | --- |
| Innate immunity and intestinal microbiota in the development of Type 1 diabetes | Nature | [Wen](https://www.webofscience.com/wos/alldb/general-summary?queryJson=%5B%7B%22rowField%22:%22AU%22,%22rowText%22:%22Wen,%20L%22%7D%5D&eventMode=oneClickSearch) | 2008 | 1442 | 90.13 |
| Sex Differences in the Gut Microbiome Drive Hormone-Dependent Regulation of Autoimmunity | Science | Markle | 2013 | 1266 | 115.09 |
| Temporal development of the gut microbiome in early childhood from the TEDDY study | Nature | [Stewart](https://www.webofscience.com/wos/alldb/general-summary?queryJson=%5B%7B%22rowField%22:%22AU%22,%22rowText%22:%22Stewart,%20CJ%22%7D%5D&eventMode=oneClickSearch) | 2018 | 878 | 146.33 |
| How informative is the mouse for human gut microbiota research | Disease models & mechanisms | [Nguyen](https://www.webofscience.com/wos/alldb/general-summary?queryJson=%5B%7B%22rowField%22:%22AU%22,%22rowText%22:%22Nguyen,%20TLA%22%7D%5D&eventMode=oneClickSearch) | 2015 | 797 | 88.56 |
| The Dynamics of the Human Infant Gut Microbiome in Development and in Progression toward Type 1 Diabetes | Cell Host & microbe | [Kostic](https://www.webofscience.com/wos/alldb/general-summary?queryJson=%5B%7B%22rowField%22:%22AU%22,%22rowText%22:%22Kostic,%20AD%22%7D%5D&eventMode=oneClickSearch) | 2015 | 714 | 79.33 |
| Variation in Microbiome LPS Immunogenicity Contributes to Autoimmunity in Humans | Cell | [Vatanen](https://www.webofscience.com/wos/alldb/general-summary?queryJson=%5B%7B%22rowField%22:%22AU%22,%22rowText%22:%22Vatanen,%20T%22%7D%5D&eventMode=oneClickSearch) | 2016 | 671 | 83.88 |
| Gender Bias in Autoimmunity Is Influenced by Microbiota | Immunity | [Yurkovetskiy](https://www.webofscience.com/wos/alldb/general-summary?queryJson=%5B%7B%22rowField%22:%22AU%22,%22rowText%22:%22Yurkovetskiy,%20L%22%7D%5D&eventMode=oneClickSearch) | 2013 | 587 | 53.36 |
| Toward defining the autoimmune microbiome for type 1 diabetes | ISME journal | [Giongo](https://www.webofscience.com/wos/alldb/general-summary?queryJson=%5B%7B%22rowField%22:%22AU%22,%22rowText%22:%22Giongo,%20A%22%7D%5D&eventMode=oneClickSearch) | 2011 | 561 | 43.15 |
| Gut microbiota in children with type 1 diabetes differs from that in healthy children: a case-control study | BMC medicine | [Murri](https://www.webofscience.com/wos/alldb/general-summary?queryJson=%5B%7B%22rowField%22:%22AU%22,%22rowText%22:%22Murri,%20M%22%7D%5D&eventMode=oneClickSearch) | 2013 | 512 | 46.55 |
| Interactions Between Gut Microbiota and Host Metabolism Predisposing to Obesity and Diabetes | Annual review of medicine | [Musso](https://www.webofscience.com/wos/alldb/general-summary?queryJson=%5B%7B%22rowField%22:%22AU%22,%22rowText%22:%22Musso,%20G%22%7D%5D&eventMode=oneClickSearch) | 2011 | 436 | 33.54 |
| The human gut microbiome in early-onset type 1 diabetes from the TEDDY study | Nature | Marino | 2018 | 430 | 71.67 |
| Gut microbial metabolites limit the frequency of autoimmune T cells and protect against type 1 diabetes | Nature immunology | [Marino](https://www.webofscience.com/wos/alldb/general-summary?queryJson=%5B%7B%22rowField%22:%22AU%22,%22rowText%22:%22Marino,%20E%22%7D%5D&eventMode=oneClickSearch) | 2017 | 428 | 61.14 |
| Environmental risk factors for type 1 diabetes | Lancet | Rewers | 2016 | 388 | 48.50 |
| Fecal Microbiota Composition Differs Between Children With beta-Cell Autoimmunity and Those Without | Diabetes | [de Goffau](https://www.webofscience.com/wos/alldb/general-summary?queryJson=%5B%7B%22rowField%22:%22AU%22,%22rowText%22:%22de%20Goffau,%20MC%22%7D%5D&eventMode=oneClickSearch) | 2013 | 386 | 35.09 |
| Role of intestinal microbiota and metabolites on gut homeostasis and human diseases | BMC immunology | Lin | 2017 | 378 | 54.00 |

###### Supplementary Table 3: The top 35 keywords with the strongest citation bursts.

| **Keywords** | **Strength** | **Begin** | **End** | **2004 - 2023** |
| --- | --- | --- | --- | --- |
| Prone rat | 2,81 | **2009** | 2014 | ▂▂▂▂▂▃▃▃▃▃▃▂▂▂▂▂▂▂▂▂ |
| Nonobese diabetic mice | 3.62 | **2010** | 2013 | ▂▂▂▂▂▂▃▃▃▃▂▂▂▂▂▂▂▂▂▂ |
| Regulatory t cells | 3.04 | **2011** | 2013 | ▂▂▂▂▂▂▂▃▃▃▂▂▂▂▂▂▂▂▂▂ |
| Innate immunity | 2.58 | **2011** | 2013 | ▂▂▂▂▂▂▂▃▃▃▂▂▂▂▂▂▂▂▂▂ |
| Animal models | 2.31 | **2011** | 2014 | ▂▂▂▂▂▂▂▃▃▃▃▂▂▂▂▂▂▂▂▂ |
| Autoimmune disease | 2.22 | **2011** | 2013 | ▂▂▂▂▂▂▂▃▃▃▂▂▂▂▂▂▂▂▂▂ |
| Differs | 3.20 | **2014** | 2016 | ▂▂▂▂▂▂▂▂▂▂▃▃▃▂▂▂▂▂▂▂ |
| Treatment partially protects | 2.56 | **2014** | 2015 | ▂▂▂▂▂▂▂▂▂▂▃▃▂▂▂▂▂▂▂▂ |
| Increased intestinal permeability | 3.61 | **2015** | 2018 | ▂▂▂▂▂▂▂▂▂▂▂▃▃▃▃▂▂▂▂▂ |
| Mice | 2.65 | **2015** | 2017 | ▂▂▂▂▂▂▂▂▂▂▂▃▃▃▂▂▂▂▂▂ |
| Delivery | 2.26 | **2015** | 2016 | ▂▂▂▂▂▂▂▂▂▂▂▃▃▂▂▂▂▂▂▂ |
| Early life | 3.09 | **2016** | 2020 | ▂▂▂▂▂▂▂▂▂▂▂▂▃▃▃▃▃▂▂▂ |
| Environmental determinants | 2.96 | **2016** | 2019 | ▂▂▂▂▂▂▂▂▂▂▂▂▃▃▃▃▂▂▂▂ |
| Commensal bacteria | 2.92 | **2016** | 2018 | ▂▂▂▂▂▂▂▂▂▂▂▂▃▃▃▂▂▂▂▂ |
| Cesarean section | 2.65 | **2016** | 2018 | ▂▂▂▂▂▂▂▂▂▂▂▂▃▃▃▂▂▂▂▂ |
| Environmental factors | 2.23 | **2016** | 2018 | ▂▂▂▂▂▂▂▂▂▂▂▂▃▃▃▂▂▂▂▂ |
| Homeostasis | 3.05 | **2017** | 2019 | ▂▂▂▂▂▂▂▂▂▂▂▂▂▃▃▃▂▂▂▂ |
| Inflammatory responses | 2.06 | **2017** | 2018 | ▂▂▂▂▂▂▂▂▂▂▂▂▂▃▃▂▂▂▂▂ |
| Autoimmunity | 3.94 | **2018** | 2019 | ▂▂▂▂▂▂▂▂▂▂▂▂▂▂▃▃▂▂▂▂ |
| Induction | 3.14 | **2018** | 2019 | ▂▂▂▂▂▂▂▂▂▂▂▂▂▂▃▃▂▂▂▂ |
| Contributes | 2.10 | **2018** | 2019 | ▂▂▂▂▂▂▂▂▂▂▂▂▂▂▃▃▂▂▂▂ |
| Dietary fiber | 2.76 | **2019** | 2021 | ▂▂▂▂▂▂▂▂▂▂▂▂▂▂▂▃▃▃▂▂ |
| Double blind | 2.29 | **2019** | 2020 | ▂▂▂▂▂▂▂▂▂▂▂▂▂▂▂▃▃▂▂▂ |
| Early childhood | 2.26 | **2019** | 2021 | ▂▂▂▂▂▂▂▂▂▂▂▂▂▂▂▃▃▃▂▂ |
| Intestinal permeability | 2.24 | **2019** | 2021 | ▂▂▂▂▂▂▂▂▂▂▂▂▂▂▂▃▃▃▂▂ |
| Progression | 3.66 | **2020** | 2023 | ▂▂▂▂▂▂▂▂▂▂▂▂▂▂▂▂▃▃▃▃ |
| Insulin sensitivity | 2.99 | **2020** | 2023 | ▂▂▂▂▂▂▂▂▂▂▂▂▂▂▂▂▃▃▃▃ |
| Diversity | 2.72 | **2020** | 2021 | ▂▂▂▂▂▂▂▂▂▂▂▂▂▂▂▂▃▃▂▂ |
| Diabetes mellitus | 2.23 | **2020** | 2021 | ▂▂▂▂▂▂▂▂▂▂▂▂▂▂▂▂▃▃▂▂ |
| Insulin resistance | 3.01 | **2021** | 2023 | ▂▂▂▂▂▂▂▂▂▂▂▂▂▂▂▂▂▃▃▃ |
| Human gut microbiome | 2.81 | **2021** | 2023 | ▂▂▂▂▂▂▂▂▂▂▂▂▂▂▂▂▂▃▃▃ |
| Glycemic control | 2.52 | **2021** | 2023 | ▂▂▂▂▂▂▂▂▂▂▂▂▂▂▂▂▂▃▃▃ |
| Butyrate | 2.45 | **2021** | 2023 | ▂▂▂▂▂▂▂▂▂▂▂▂▂▂▂▂▂▃▃▃ |
| Faecalibacterium prausnitzii | 2.11 | **2021** | 2023 | ▂▂▂▂▂▂▂▂▂▂▂▂▂▂▂▂▂▃▃▃ |
| Blood glucose | 2.11 | **2021** | 2023 | ▂▂▂▂▂▂▂▂▂▂▂▂▂▂▂▂▂▃▃▃ |

**Note:** Black bars indicated that the keyword appeared frequently. Gray bars indicated that the keyword appeared rarely. The greater the intensity, the higher the frequency.
